# Molecular Profiling and Gene Banking of Rabbit EPCs Derived from Two Biological Sources

**DOI:** 10.3390/genes12030366

**Published:** 2021-03-04

**Authors:** Jaromír Vašíček, Andrej Baláži, Miroslav Bauer, Andrea Svoradová, Mária Tirpáková, Marián Tomka, Peter Chrenek

**Affiliations:** 1 NPPC, Research Institute for Animal Production Nitra, Institute of Farm Animal Genetics and Reproduction, Hlohovecká 2, 951 41 Lužianky, Slovakia; andrej.balazi@nppc.sk (A.B.); miroslav.bauer@nppc.sk (M.B.); andrea.svoradova@nppc.sk (A.S.); marian.tomka@uniag.sk (M.T.); 2 Department of Biochemistry and Biotechnology, Faculty of Biotechnology and Food Science, Slovak University of Agriculture in Nitra, Tr. A. Hlinku 2, 949 76 Nitra, Slovakia; maria.tirpakova@uniag.sk; 3 Department of Botany and Genetics, Faculty of Natural Sciences, Constantine the Philosopher University in Nitra, Nábrežie mládeže 91, 949 74 Nitra, Slovakia; 4 AgroBioTech Research Center, Slovak University of Agriculture in Nitra, Tr. A. Hlinku 2, 949 76 Nitra, Slovakia

**Keywords:** rabbit, peripheral blood, bone marrow, endothelial progenitor cells, HUVECs, flow cytometry, qPCR, ddPCR, neuro-transdifferentiation, cryopreservation

## Abstract

Endothelial progenitor cells (EPCs) have been broadly studied for several years due to their outstanding regenerative potential. Moreover, these cells might be a valuable source of genetic information for the preservation of endangered animal species. However, a controversy regarding their characterization still exists. The aim of this study was to isolate and compare the rabbit peripheral blood- and bone marrow-derived EPCs with human umbilical vein endothelial cells (HUVECs) in terms of their phenotype and morphology that could be affected by the passage number or cryopreservation as well as to assess their possible neuro-differentiation potential. Briefly, cells were isolated and cultured under standard endothelial conditions until passage 3. The morphological changes during the culture were monitored and each passage was analyzed for the typical phenotype using flow cytometry, quantitative real–time polymerase chain reaction (qPCR) and novel digital droplet PCR (ddPCR), and compared to HUVECs. The neurogenic differentiation was induced using a commercial kit. Rabbit cells were also cryopreserved for at least 3 months and then analyzed after thawing. According to the obtained results, both rabbit EPCs exhibit a spindle-shaped morphology and high proliferation rate. The both cell lines possess same stable phenotype: CD14^−^CD29^+^CD31^−^CD34^−^CD44^+^CD45^−^CD49f^+^CD73^+^CD90^+^CD105^+^CD133^−^CD146^−^CD166^+^VE-cadherin^+^VEGFR-2^+^SSEA-4^+^MSCA-1^−^vWF^+^eNOS^+^AcLDL^+^ALDH^+^vimentin^+^desmin^+^α-SMA^+^, slightly different from HUVECs. Moreover, both induced rabbit EPCs exhibit neuron-like morphological changes and expression of neuronal markers ENO2 and MAP2. In addition, cryopreserved rabbit cells maintained high viability (>85%) and endothelial phenotype after thawing. In conclusion, our findings suggest that cells expanded from the rabbit peripheral blood and bone marrow are of the endothelial origin with a stable marker expression and interesting proliferation and differentiation capacity.

## 1. Introduction

The banking of stem and progenitor cells is a common method used to cryogenically preserve those cells for their further therapeutical or other biomedical use. These cell banks contain cells isolated from various sources such as bone marrow, peripheral blood, umbilical cord blood or tissue, adipose tissue and/or other tissue types [1]. However, a proper methodology is required in order to isolate and characterize the cells that will be stored in the cell banks of human or even other animal’s origin. In addition, animal cell banks are part of the gene banks that preserve the genetic information of different livestock breeds, and thus protect global animal biodiversity [2].

Generally, somatic stem cells are known to possess three basic properties: self-renewability, clonogenicity, and differentiation capacity (plasticity) [3]. These cells generate a replacement progeny for the organ or tissue in which they are resided within the nurturing niches. Similarly, the progenitor cells provide mature cells for the tissues in which they are mostly located. On the other hand, although they may clonally expand prior to their differentiation into mature cells, they lack self-renewal potential [4].

Endothelial progenitor cells (EPCs), firstly isolated from human peripheral blood [5], seem to be unique, since they are closely similar to stem cells in terms of self-renewability, clonogenicity, and their plasticity. Moreover, they might be defined as unipotent stem cells that are able to uptake acetylated low-density lipoproteins (acLDL), to bind *Ulex europaeus* agglutinin-1 (UEA-1) and to take part in the neovascularization [3]. Two different types of EPCs have been recognized in human until now, early EPCs and late EPCs. Their morphologies, time of appearance, and protein expression have been described in several studies [6,7,8,9]. Over two decades of EPCs research has revealed that beside the peripheral blood they can be isolated and/or transdifferentiated from other sources such as bone marrow, myeloid cells or even mesenchymal stem cells, umbilical cord blood or tissue, and adipose, cardiac, neural or dental tissue etc., while maintaining similar phenotypic characteristics [3]. There are three common methods for the isolation of EPCs from peripheral blood that can be also applied for bone marrow. The first one is the direct isolation of EPCs using magnetic- or fluorescent-activated cell sorting (MACS or FACS, respectively) based on the specific marker expression [5,10,11,12,13,14]. The next one and the most used method is the depletion technique, when mononuclear cells (MNCs) are plated on the dishes and cultured approximately for 4 days. Then, nonadherent cells (platelets, red blood cells or monocytes) are removed (depleted) by washing with phosphate-buffered saline (PBS). After 6–7 days, spindle-shaped cells appear in the culture (early EPCs). On the other hand, cobblestone cells are visible after four weeks of culture (late EPCs) [6,7,15,16]. The third method, named colony-forming unit Hill assay, is a replating technique, in which the cells that did not adhere after the plating of MNCs are replated again after 24 or 48 h. However, this method is not preferable according to its variable results [17,18]. Nevertheless, the identification of the EPCs is still controversial mainly due to a lack of standardization in their isolation and characterization [19]. Altogether, the early EPCs are reported to express progenitor markers as CD34 and CD133 as well as VEGFR-2 (Flk-1/KDR), while the late EPCs lose the expression of CD34 and CD133 and express endothelial-associated markers such as von Willebrand factor (vWF), CD31, VE-cadherin (CD144), endothelial nitric oxide synthase (eNOS), VEGFR-2, CD105 and CD146 [3,5,8,13,20,21,22,23,24,25]. Moreover, the recent study [26] demonstrated that late EPCs possess similar phenotype (CD31^+^vWF^+^KDR^+^CD146^+^CD34^−^CD133^−^CD45^−^CD90^−^) as human umbilical vein endothelial cells (HUVECs). Interestingly, a transdifferentiation of HUVECs into neuron-like cells was observed under certain culture conditions [27,28,29], although there is no information about such differentiation potential of EPCs.

Beside the human model, EPCs have been already isolated from the peripheral blood and/or bone marrow of mouse [30], rat [31,32,33,34,35], dog [36,37], sheep [22] and goat [38] or even chicken [39]. Moreover, the EPCs were isolated also from the peripheral blood and bone marrow of rabbits more than ten years ago [40,41,42,43,44]. However, their phenotypic analysis, although compared to HUVECs, includes only few selected endothelial cell markers, expression of which is strongly variable among those studies. On the other hand, cells intended for the gene banking should be analyzed for their stable phenotype and function during the whole culture that should not be affected by their storage in the liquid nitrogen. Therefore, the aim of this study was to isolate and compare the rabbit peripheral blood- and bone marrow-derived EPCs with HUVECs in terms of their phenotype and morphology that could be affected by the passage number or cryopreservation as well as to assess their possible neuro-differentiation potential.

## 2. Materials and Methods

### 2.1. Animals

Clinically healthy and young (3- to 8-month-old) rabbits (*n* = 20) of the New Zealand White (NZW) line reared as described previously [45] were used in this study. The treatment of the animals was approved by the Ministry of Agriculture and Rural Development of the Slovak Republic no. SK U 18016 in accordance with the ethical guidelines presented in Slovak Animal Protection Regulation (RD 377/12), which conforms to the Code of Ethics of the EU Directive 2010/63/EU for animal experiments.

### 2.2. Experimental Design

The experiments were designed in order to isolate, characterize and cryopreserve the pure cell population of rabbit endothelial progenitor cells, which were derived from peripheral blood (EPCs) and bone marrow (BEPCs). Specific features of the both rabbit cell lines were compared between each other as well as with the specific primary HUVECs. Typical cell morphology and phenotype were observed and compared among all above-mentioned cell lines. Moreover, a neurogenic differentiation assay was applied for both rabbit and human cell lines. Finally, the rabbit EPCs and BEPCs were cryopreserved and stored in liquid nitrogen for at least 3 months. Thereafter, randomly selected samples from both rabbit cell lines were thawed in order to check their viability and proliferation ability as well as their phenotype.

### 2.3. Collection of Biological Material and Isolation of Rabbit Endothelial Progenitor Cells

Mononuclear cells from peripheral blood (PBMCs) and bone marrow (BMMCs) collected from humanely sacrificed rabbits were isolated using Biocoll (Biochrom, Berlin, Germany) density-gradient centrifugation as described previously [2,46]. Briefly, cells were then resuspended in endothelial growth (EGM-2) medium that contained endothelial basal (EBM-2) basal medium, recombinant growth factors: bFGF, EGF, R3-IGF-1 and vitamin C (Lonza, Walkersville, MD, USA); 20% fetal calf serum (Biowest, Riverside, MO, USA) and penicillin/streptomycin solution (Thermo Fisher Scientific, Waltham, MA, USA). Cells were plated at the density of 0.4–0.5 × 10^6^ cells/cm^2^ into T25 or T75 tissue culture flasks. Cultures were maintained at 37 °C in a 5% CO_2_ humidified atmosphere. Following 3 days of the culture, non-adherent cells were discarded by changing the medium for the fresh one. Culture medium was changed every 3–4 days until the adherent cells reached the confluency of 80–90% (passage 0; P0). Then, after washing with PBS (without Ca and Mg; Biowest, Riverside, MO, USA), cells were harvested using 0.05% Trypsin-EDTA (Thermo Fisher Scientific, Waltham, MA, USA) and counted using EVE™ Automatic cell counter (NanoEntek, Seoul, Korea). The cells were reseeded as a subsequent passage at the concentration of 2 × 10^6^/T75. This process was repeated until the passage 3. Cell aliquots from each passage (P0–P3) were used for the phenotyping of cells using flow cytometry and polymerase chain reaction (PCR) methods. Moreover, during the culture cell morphology was observed using Zeiss Axio Observer.Z1/7 microscope (Carl Zeiss Slovakia, Bratislava, Slovakia). In addition, doubling time analyses of both rabbit cell lines were performed as described previously [47].

### 2.4. Culture of Human Endothelial Cell Line

The HUVECs cell line was obtained from ATCC (Primary Umbilical Vein Endothelial Cells; Normal, Human (HUVEC) (ATCC^®^ PCS-100-010™)). Frozen cells (passage 1) were thawed in water bath and seeded at the density of 2.5–5 × 10^4^/cm^2^ according to the producer’s manual. The aforementioned EGM-2 medium was used for the initial culture and additional subculturing until the passage 4. Morphology and doubling time analysis were performed as mentioned for rabbit cells. Furthermore, the typical phenotype of these cells was observed using flow cytometry in passage 3.

### 2.5. Flow Cytometry

**Figure A1:**
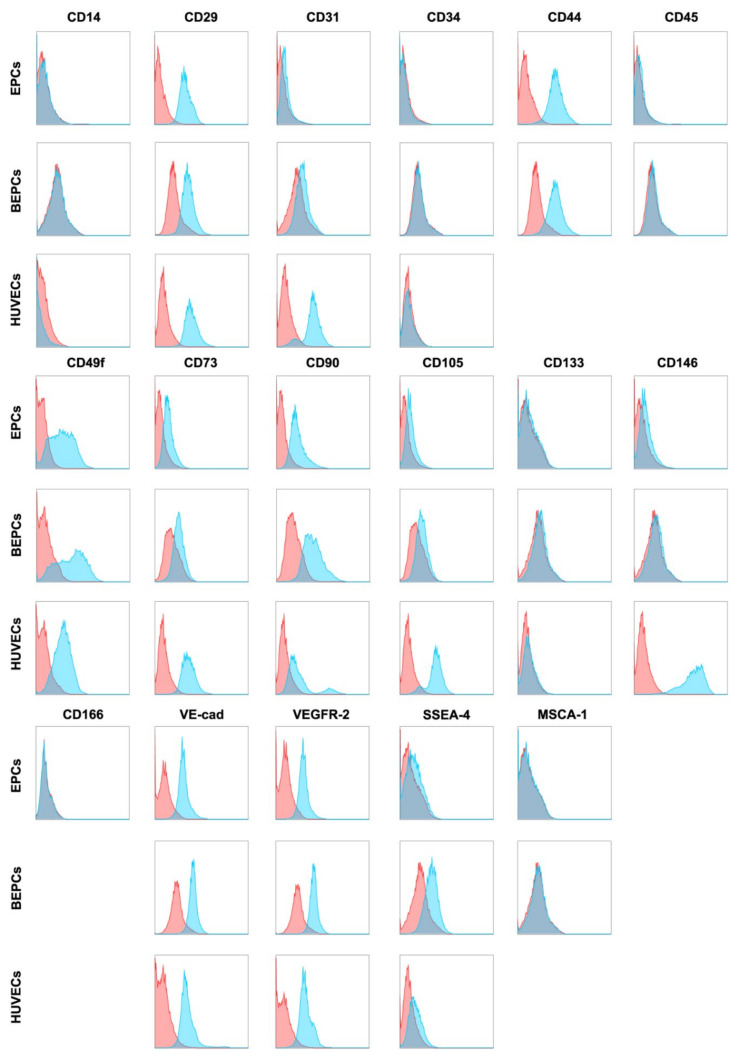
Illustrative flow-cytometric histogram plots comparing expression of surface markers by rabbit and human cells (passage 3). Red peak: Fluorescence minus one (FMO) unstained control, Blue peak: antibody staining of specific marker.

**Figure A2:**
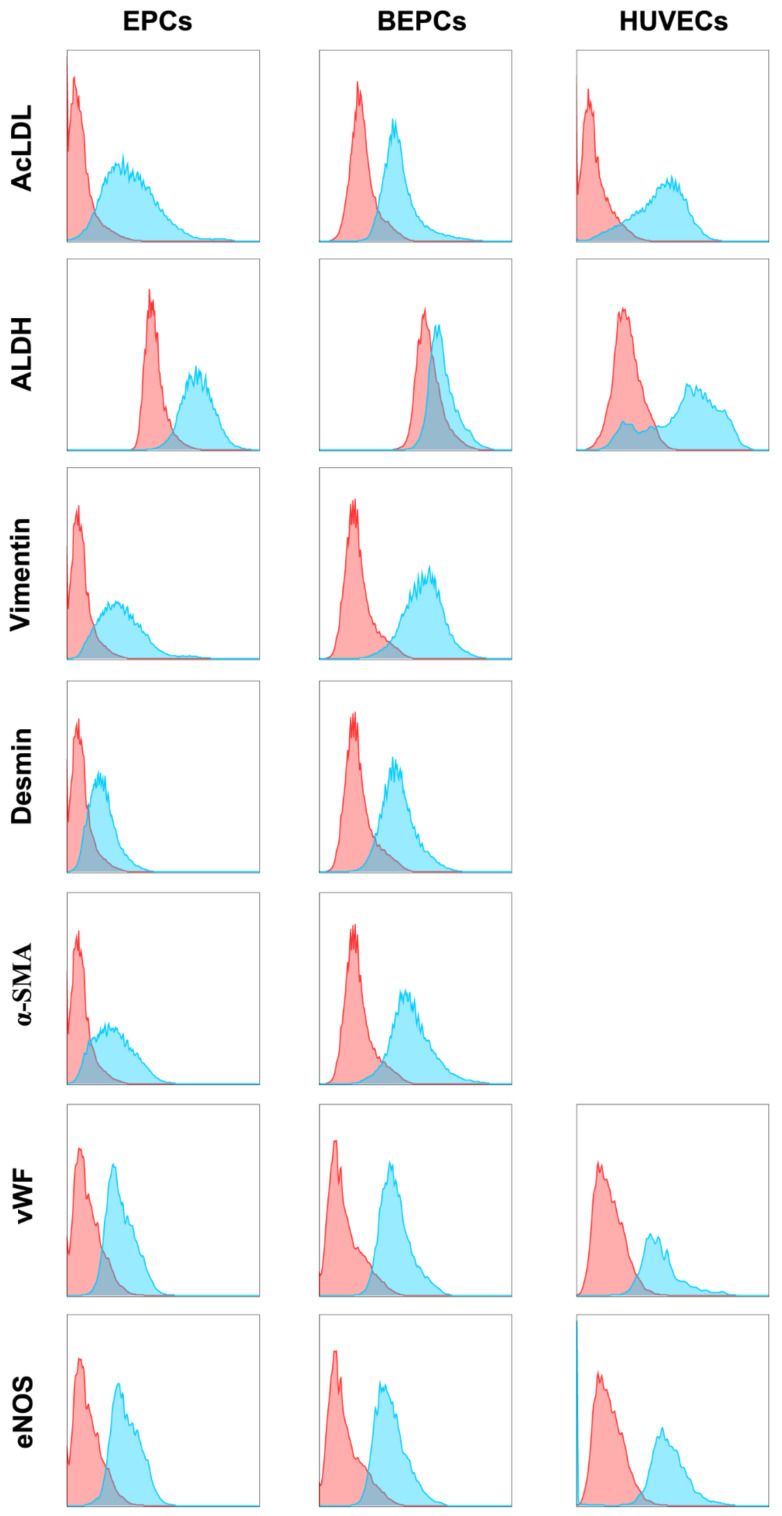
Illustrative flow-cytometric histograms comparing expression of intracellular markers by rabbit and human cells (passage 3). Red peak: Fluorescence minus one (FMO) unstained control, Blue peak: antibody staining of specific marker.

The changes in the phenotypic expression of membrane (CD14, CD29, CD31, CD34, CD44, CD45, CD49f, CD73, CD90, CD105, CD133, CD146, CD166, VE-cadherin, VEGFR-2, SSEA-4 and MSCA-1) and intracellular markers (vimentin, desmin, α-SMA (α–smooth muscle actin), von Willebrand factor (vWF) and endothelial nitric oxide synthase (eNOS)) during the culture and passaging (P0–P3) of both rabbit endothelial cell lines were observed using flow cytometry. The expression of selected markers was also analyzed in the third passage of HUVECs. Staining of cells with the primary antibodies was performed as described previously [45]. The proper secondary antibodies were used for the purified primary antibodies: rat anti-mouse IgG1-FITC (clone M1-14D12; eBioscience, Wien, Austria), goat anti-rabbit IgG-FITC (polyclonal; 405002, Bio-Rad, Hercules, CA, USA) and goat anti-mouse IgG-FITC (polyclonal; STAR117F, Bio-Rad, Hercules, CA, USA). The list of primary antibodies that were used in this study is shown in Table 1. The illustrative flow-cytometric plots are shown in Appendix A (Figure A1 and Figure A2). The primary antibodies used were anti-human with declared or expected cross-reactivity to rabbit according to the homology of rabbit and human markers (Appendix B: Table A1).

Since endothelial cells are characterized by endocytosis of acetylated low-density lipoprotein (AcLDL), rabbit EPCs and BEPCs (P0–P3) as well as HUVECs (P3) were incubated with Alexa Fluor 488 AcLDL (Molecular Probes, Eugene, OR, USA) at a concentration of 2 μg/mL for 2 h at 37 °C. In addition, the activity of aldehyde dehydrogenase (ALDH) in the aforementioned cells was analyzed using ALDEFLUOR^TM^ kit (STEMCELL Technologies, Vancouver, Canada) as described previously [48].

In order to check the viability and apoptosis of rabbit EPCs and BEPCs in the initial culture and additional passages (P0–P3), an Annexin V Apoptosis Detection Kit containing Annexin V-FITC and propidium iodide (Canvax, Cordoba, Spain) was used according to the producer’s manual. The subsequent cell populations were assessed using flow cytometry: unstained (live) cells (AnV^−^/PI^−^), apoptotic cells (AnV^+^/PI^−^) and dead cells (AnV^+^/PI^+^ and AnV^−^/PI^+^).

The stained samples were analyzed by a FACSCalibur flow cytometer (BD Biosciences, San Jose, CA, USA) that acquired 10,000–50,000 cells per samples depending on the analysis.

### 2.6. Polymerase Chain Reaction (PCR) Analyses

Total RNA from each passage (P0–P3) of rabbit EPCs and BEPCs was isolated and cDNA synthesis was performed as described previously [47]. Primers used in this study for PCR analyses (Table 2) have been either already published or designed de novo using the Primer-BLAST at the NCBI website [49].

#### 2.6.1. Real-Time PCR

A quantitative real–time PCR (qPCR) was used to monitor changes in the mRNA expression of several markers among the different passages of both rabbit cell lines. Markers that were published to be expressed by endothelial cells (CD31, CD34, CD105, CD133, CD146 and CD166) or markers (SSEA-4, MSCA-1 and ALDH) the expression of which should be confirmed by other methods were selected for qPCR. The reaction with some modifications was performed as described previously [47]: initial denaturation and activation of hot-start DNA polymerase at 95 °C for 7 min followed by 40 cycles of denaturation at 95 °C for 10 s, annealing at 63 °C for 10 s and extension at 72 °C for 15 s in Rotor-Gene 6000 (Corbett Research, Sydney, Australia). A relative quantification of gene expression to housekeeping gene β-2-microglobulin (B2M) was calculated using the threshold (CT) values and PCR reaction efficiencies according to Pfaffl [51].

#### 2.6.2. Droplet Digital PCR

In order to absolutely quantify the expression of selected markers (CD31, CD34, CD73, CD90, CD105, CD133, CD146, CD166, SSEA-4 and MSCA-1), detection of which by flow cytometry seemed to be doubtful due to the weak specificity of the used antibody, a novel digital droplet PCR (ddPCR) method was used. Highly expressed (CD29 and CD44) or lineage-negative markers (CD45) were used as control markers for comparing the efficiency of ddPCR with flow cytometry to quantify the marker expression.

Briefly, the reaction mixture at the final volume of 20 μL contained 10 μL of QX200™ ddPCR™ EvaGreen Supermix (Bio-Rad, Hercules, CA, USA), 1 μL of cDNA, 0.5 μL of primers and 8 μL of ultrapure water. This volume was subsequently mixed with 70 μL of QX200^TM^ Droplet Generation Oil for EvaGreen system (Bio-Rad, Hercules, CA, USA) in order to make droplets using a QX200™ Droplet Generator (Bio-Rad, Hercules, CA, USA) according to the producer’s manual. Then, the droplet mixtures were applied into the 96-well PCR plate (Bio-Rad, Hercules, CA, USA) and sealed prior the PCR reaction. The PCR reaction was performed in a T100 thermal cycler (Bio-Rad, Hercules, CA, USA) under the following conditions: initial denaturation and activation of hot-start DNA polymerase at 95 °C for 2 min followed by 40 cycles of denaturation at 95 °C for 15 s, annealing at 60 °C for 15 s and extension at 72 °C for 15 s. All samples were amplified in duplicate. After amplification, the samples were analyzed by a QX200 Droplet Reader and evaluated using Quanta Soft version 1.7.4.0917 (both from Bio-Rad, Hercules, CA, USA). Only samples containing at least 12,000 droplets were used for the quantification, in order to achieve the most accurate results. The expression of selected markers quantified by the ddPCR method was recalculated to the percentages as the proportion of the number of positive droplets to the total number of droplets in the sample.

### 2.7. Cell Differentiation into the Neurogenic Lineage

In order to assess the possible neurogenic potential rabbit endothelial progenitor cells (EPCs and BEPCs) as well as human HUVECs, cells at passage three (both rabbit) and four (human) and at the confluency of 60–80% were incubated in the commercial medium for the neurogenic differentiation of cells (Mesenchymal Stem Cell Neurogenic Differentiation Medium; PromoCell, Heidelberg, Germany) according to the producer’s manual. After 3 days of culture, the cell morphology was observed as mentioned before and cells were harvested for the qPCR analysis. In addition, fluorescent confocal microscopy of the induced cells was also performed.

#### 2.7.1. Quantitative Real–Time PCR (qPCR) Analysis of Neuronal Markers

Total RNA isolation and cDNA synthesis from the induced and control (uninduced) samples were carried out as described before. Two typical neuronal markers, neuron-specific enolase (ENO2) and microtubule associated protein 2 (MAP2), were used in order to confirm the neurodifferentiation of endothelial cells. The specific primers for rabbit markers were designed de novo using the Primer-BLAST at the NCBI website [49] as follows: ENO2 (5´-3´, ACACACTCAAGGGGGTCATC; GTCGATGGCTTCCTTTACCA, accession No. XM_002712914.3) and MAP2 (5´-3´, CTCACCATGTTCCTGGAGGT; GGAGGAGACGTTGCTGAGTC, accession No. XM_017343068.1). The above mentioned rabbit B2M (Table 2) was used as the reference gene. For HUVECs, published human specific primers [52] were used as follows: ENO2 (5´-3´, GGAGAACAGTGAAGCCTTGG; GGTCAAATGGGTCCTCAATG), MAP2 (5´-3´, AGTTCCAGCAGCGTGATG; CATTCTCTCTTCAGCCTTCTC) and human actin (ACT) (5´-3´, CCTGGCGTCGTCATTAGTG; TCAGTCCTGTCCATAATTAGTCC) as the reference gene. The PCR amplification and relative quantification of gene expression were performed as described before under the following conditions: an initial denaturation and activation of Taq DNA polymerase of 95 °C for 7 min followed by 40 cycles of 95 °C for 10 s, 60 °C for 10 s and 72 °C for 10 s.

#### 2.7.2. Expression of the Neuronal Markers Assessed by Confocal Microscopy

Briefly, neuro-differentiated cells (rabbit and human) cultured on the round coverslips at the bottom of the Nunc™ 4-well dishes (Thermo Fisher Scientific, Waltham, MA, USA) were fixed using IC Fixation Buffer (Thermo Fisher Scientific, Waltham, MA, USA) for 20 min. and subsequently permeabilized and blocked using 0.1% Triton X-100 and 1% bovine serum albumin (BSA) solution in PBS for 30 min. on ice. After washing, coverslips with cells were incubated with the purified mouse monoclonal antibodies against ENO2 (clone NSE47; Enzo Life Sciences, Farmingdale, NY, USA) and MAP2 (clone BB7; Creative Diagnostics, Shirley, NY, USA) overnight. On the other day, coverslips were washed and incubated with polyclonal goat anti-mouse FITC-conjugated secondary antibody (STAR117F; Bio-Rad, Hercules, CA, USA) for 15 min. on ice. After the final wash, 4 μL of the VECTASHIELD HardSet antifade mounting medium with DAPI (Vector Laboratories, Burlingame, CA, USA) was dropped on the microscope slide and each coverslip was carefully mounted facing the cells towards the microscope slide. Prepared samples were analyzed using the laser scanning confocal microscope LSM 700 (Carl Zeiss Slovakia, Bratislava, Slovakia).

### 2.8. Cryopreservation of the Rabbit Endothelial Progenitor Cells (EPCs)

Rabbit EPCs and BEPCs at the passage 3 (3–5 × 10^6^ cells per cryovial) were resuspended in the freezing medium composed of culture medium (EGM-2 medium with 20% of fetal calf serum) supplemented with 10% of dimethyl sulfoxide (DMSO, EMPLURA; Merck, Darmstadt, Germany). Cryovials were then placed into freezing container (Mr. Frosty; Thermo Scientific Nalgene, Rochester, NY, USA) and stored for 24 h at −80 °C. On the following day, samples were transferred into liquid nitrogen and stored at least for 3 months. After this period, randomly chosen samples were thawed as described previously [2]. To evaluate the efficiency of cryopreservation protocol used for the freezing of the rabbit EPCs and BEPCs, part of the thawed cells was assessed for their viability using flow cytometry as mentioned before. The rest of the cells were plated again into the T75 culture flasks as passage 4 in order to evaluate their proliferation ability after cryopreservation. Those cells were cultured as described before in the Methods until the confluency of 80–90%. Then, the viability of harvested cells was analyzed for the last time as well as their typical endothelial phenotype (CD29^+^, CD44^+^, CD45^−^, VE-cadherin^+^ and VEGFR-2^+^) using flow cytometry as described before.

### 2.9. Statistical Analysis

Data obtained from analyses were evaluated using GraphPad Prism version 9.0.0 for Windows (GraphPad Software, San Diego, CA, USA) with one-way analysis of variance (ANOVA)–multiple comparisons tests (Tukey test for doubling time and Dunnett’s test for phenotype and viability analyses) and two-way ANOVA (Fisher’s least significant difference (LSD) test) for neuronal markers. Results are expressed as the mean ± SD. *p*-values at *p* < 0.05 were considered as statistically significant.

## 3. Results

### 3.1. Cell Morphology, Proliferation and Viability

**Figure 1:**
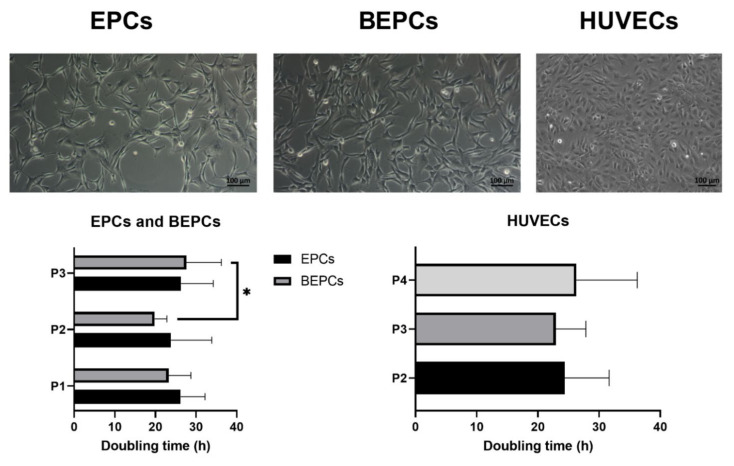
Cell morphology and population doubling time of the cultured endothelial progenitor cells. Upper part: Rabbit endothelial progenitor cells derived from peripheral blood (endothelial progenitor cells (EPCs)) as well as those derived from bone marrow (BEPCs) showed a spindle-shaped morphology (passage 3), whereas human umbilical vein endothelial cells (HUVECs, passage 4) had a cobblestone appearance as observed under a Zeiss Axio Observer.Z1/7 microscope (magnification at 100×; scale bar = 100 μm). Lower part: Both rabbit (EPCs and BEPCs) as well as HUVEC cell cultures were able to double their concentration after 20–30 h of culture independently of the passage number. The data are expressed as the mean ± standard deviation (SD); *—difference is statistically significant at *p* < 0.05.

Both rabbit endothelial cell lines (EPCs and BEPCs) required no more than 2 weeks of culture in order to reach the homogenous population with the confluency of at least 90% (passage 0). After that, the cells were able to double their number in the following passages each 20–30 h of the culture. Moreover, their proliferation capacity was not changed among the individual passages (P1-P3), except the passage 3 of BEPCs that proliferate a little bit slower (*p* > 0.05) than the previous passage 2 (Figure 1). In summary, their population doubling time was comparable to HUVECs, which needed similarly about 25 h in order to double their number independently of the cultured passage. During the whole culture the pure populations of EPCs and BEPCs kept the spindle-shaped morphology. On the other hand, HUVECs showed a typical cobblestone appearance all over the culture (Figure 1).

The viability of rabbit EPCs and BEPCs remained satisfactory during the culture (P0–P3) as the percentage of live cells varied from 84% to 92% in case of EPCs or from 89% to 93% in case of BEPCs (Table 3). Moreover, the proportion of apoptotic cells in the culture was negligible (less than 1% for EPCs and no more than 2% for BEPCs) or low in case of dead cells (no more than 15% for EPCs and less than 10% for BEPCs).

### 3.2. Phenotypic Analyses of the Primary Endothelial Cells

#### 3.2.1. Immunophenotyping

The antibody staining and flow-cytometric analyses revealed that both rabbit cell lines (EPCs and BEPCs) at the passage 3 were positive for the several membrane markers such as CD29, CD44, CD49f, CD73, CD90, CD105, VE-cadherin and VEGFR-2 (Table 4). On other hand, cells at this passage were negative or very dim positive for CD14, CD31, CD45, CD133, CD146 or CD166. Interestingly, BEPCs were partially positive for SSEA-4 (stage specific embryonic antigen-4), whereas EPCs did not express this marker according to the flow cytometry analysis. Moreover, both of them were also negative for MSCA-1 (mesenchymal stem cell antigen-1). The HUVECs (passage 3) expressed very similar phenotype: CD29^+^CD49f^+^CD73^+^CD90^+/low^CD105^+^VE-cadherin^+^VEGFR-2^+^. However, only 12% of HUVECs were positive for CD90, while about 90% of rabbit cells expressed this marker. On the contrary, more than 80% of HUVECs were also positive for CD31 and CD146, whereas both rabbit cell lines were dim positive or negative for those markers. In addition, HUVECs were negative for CD14, CD34, CD133 and SSEA-4.

The phenotypic expression of some membrane markers analyzed by flow cytometry was changing during the culture. The expression of CD14, CD31 and CD45 in both rabbit cell cultures decreased significantly in P1, P2 and P3 in comparison to the initial culture (P0), while CD29 and VE-cadherin positivity increased significantly in the subsequent passages compared to P0, although only in EPCs (Table 4). Interestinly, a very variable SSEA-4 positivity was observed in BEPCs, which had a decreasing tendency in the following passages. The phenotypic expression of the other markers among the individual pasassages did not change significantly or were not analyzed (Table 4).

In case of intracellular markers, the third passage of both rabbit cell lines (EPCs and BEPCs) exhibit a high expression (>80%) of all analyzed markers (acetylated low-density lipoprotein (AcLDL), aldehyde dehydrogenase (ALDH), vimentin, desmin, α-smooth muscle actin (α-SMA), von Willebrand factor (vWF) and endothelial nitric oxide synthase (eNOS)) with few exceptions for ALDH (only 25% in BEPCs) and for desmin (about 64% in EPCs; Table 5). The high expression (over 80%) of selected intracellular markers (AcLDL, ALDH, vWF and eNOS) was observed also in HUVECs (passage 3). Concerning the phenotype of individual passages (P0–P3) of rabbit cell lines, a significant decrease of AcLDL in the P3 of BEPCs in comparison to P0 was observed. Although the expression was still over 97%. Additionally, vWF increased significantly in the following passages when compared to P0 (for both, EPCs and BEPCs). No other significant changes in the expression of the aforementioned markers were noticed among the passages (Table 5).

#### 3.2.2. qPCR Analysis

**Figure 2:**
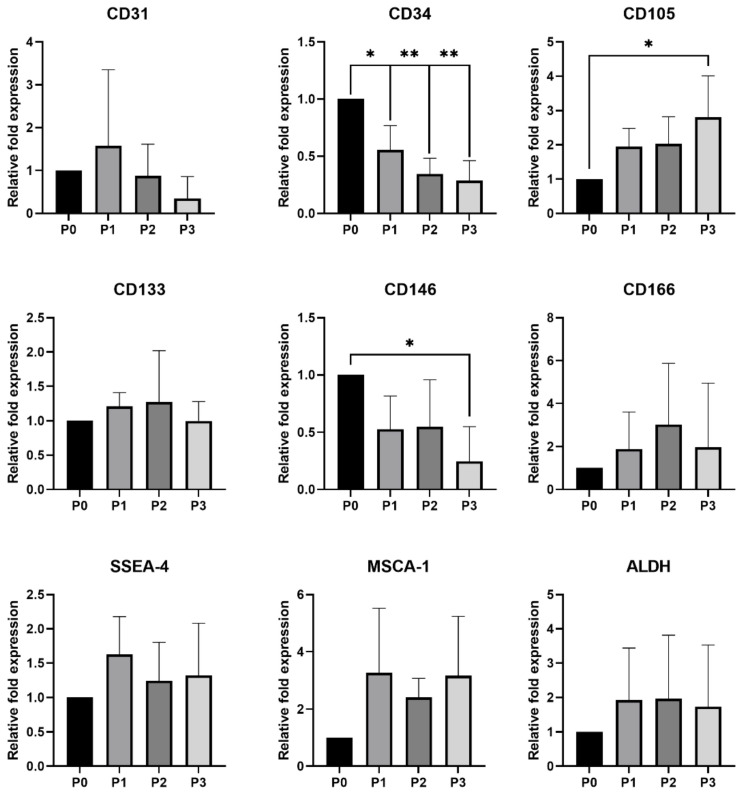
Relative expression of selected markers compared among the different passages of rabbit EPCs. P0—initial culture, P1—first passage, P2—second passage, P3—third passage. The data are expressed as the mean ± SD; *—difference is statistically significant at *p* < 0.05; **—difference is statistically significant at *p* < 0.01.

**Figure 3:**
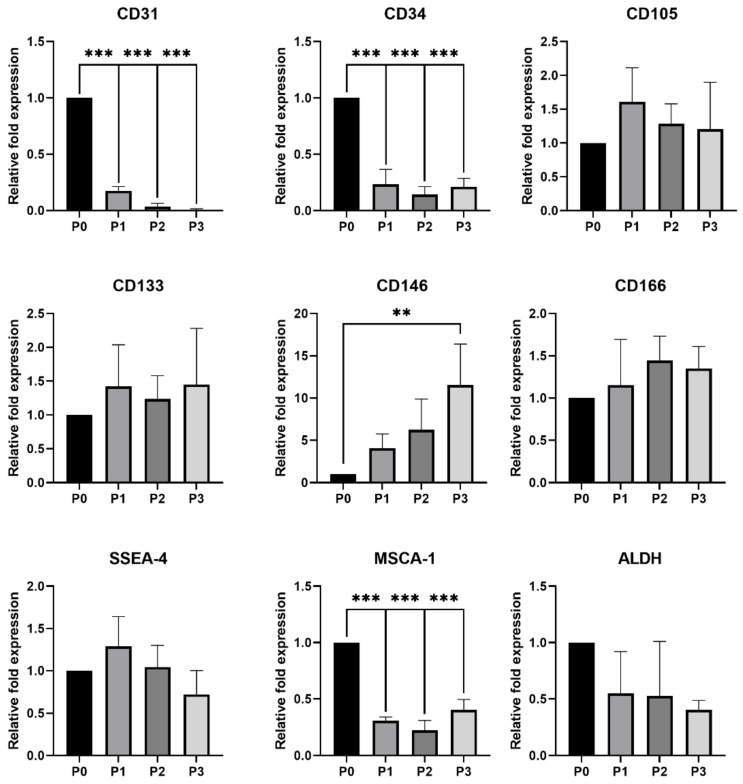
Relative expression of selected markers compared among the different passages of rabbit BEPCs. P0—initial culture, P1—first passage, P2—second passage, P3—third passage. The data are expressed as the mean ± SD; **—difference is statistically significant at *p* < 0.01; ***—difference is statistically significant at *p* < 0.001.

A relative expression of the endothelial markers expression of which was not detected using flow cytometry (CD31, CD34, CD105, CD133, CD146 and CD166) were analyzed and detected among the individual passages (P0–P3) of both rabbit cell lines (Figure 2 and Figure 3). Moreover, the relative expression of other selected markers (SSEA-4, MSCA-1 and ALDH) was also confirmed. In both, rabbit EPCs and BEPCs, a significant decrease in the CD34 relative expression was noticed in the following passages (P1-P3) when compared to the initial culture (P0). Another significant decrease of CD31’s relative expression was noticed in BEPCs, while this decrease was not significant in EPCs. Similarly, the relative expression of MSCA-1 significantly decreased in subsequent passages of BEPCs after P0 (Figure 3), although not in EPCs. On the other hand, CD105’s relative expression significantly (*p* < 0.05) increased in P3 of EPCs compared to P0 (Figure 2), although no differences in this marker expression were observed among the passages of BEPCs. Interestingly, P3 of EPCs exhibited significantly (*p* < 0.05) lower relative expression of CD146 compared to P0, whereas the same passage of BEPCs showed a significant (*p* < 0.01) rise of its expression in comparison to P0. No more significant changes in the relative expression of the other analyzed markers were detected among the passages of both rabbit cell lines.

#### 3.2.3. Digital Droplet PCR (ddPCR) Analysis

**Figure 4:**
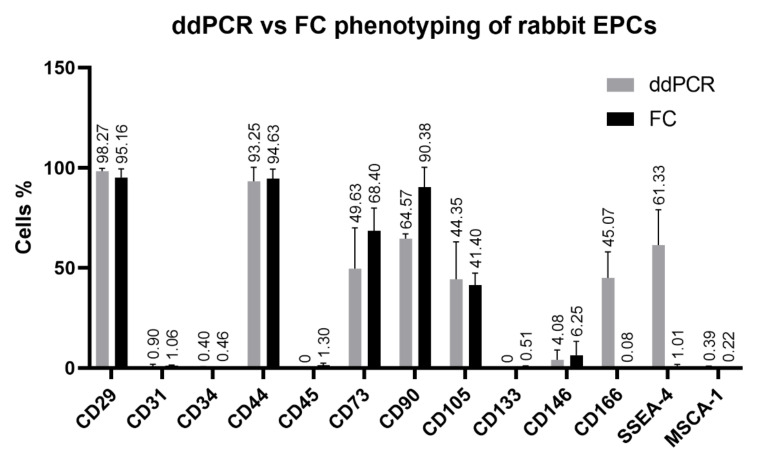
Comparison of the rabbit EPCs phenotyping using two different biological methods: droplet digital PCR and flow cytometry. ddPCR—droplet digital PCR, FC—flow cytometry. The data are expressed as the mean ± SD.

**Figure 5:**
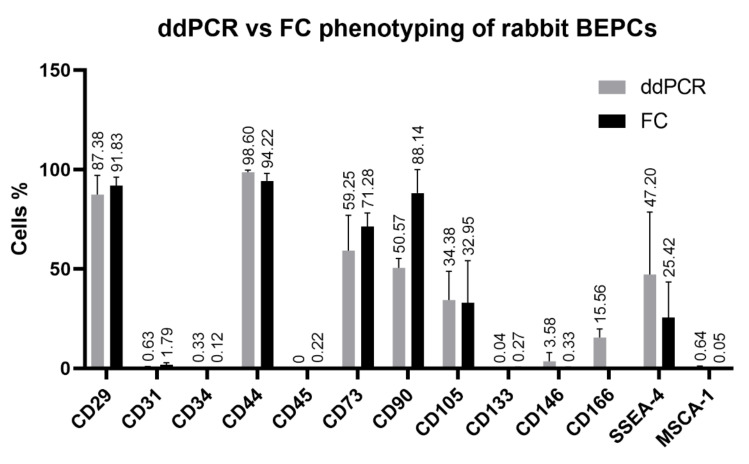
Comparison of the rabbit BEPCs phenotyping using two different biological methods: droplet digital PCR and flow cytometry. ddPCR—droplet digital PCR, FC—flow cytometry. The data are expressed as the mean ± SD.

Several membrane markers (CD29, CD31, CD34, CD44, CD45, CD73, CD90, CD105, CD133, CD146, CD166, SSEA-4 and MSCA-1) analyzed by flow cytometry and/or qPCR were selected in order to assess the efficiency of ddPCR method to confirm and quantify their expression in the third passage of both rabbit cell lines. The high expression of CD29 and CD44 detected in both, rabbit EPCs and BEPCs, using flow cytometry was confirmed also by ddPCR (Figure 4 and Figure 5) as well as no expression of CD45. Further, ddPCR revealed really low or negligible positivity for CD31 (<2%) or for CD34, CD133 and MSCA-1 (<0.5%), which agreed with the flow-cytometric values. On the contrary, similar values as detected by flow cytometry were noticed using ddPCR for CD73 (70 vs. 50% for EPCs and 60 vs. 70% for BEPCs) and for CD105 (about 40 % for EPCs and about 30% for BEPCs as detected by both methods). In case of CD90 expression, lower values were found with ddPCR compared to flow cytometry in EPCs and BEPCs (65 vs. 90% and 50 vs. 88%, respectively). Low positivity (about 4%) of CD146 was detected in the both lines, and thus confirmed the 6% positivity for EPCs as detected by flow cytometry (Figure 4). However, BEPCs did not express CD146 when evaluated using flow cytometry (Figure 5). Despite the fact that flow cytometry did not detect the CD166 expression, ddPCR revealed positivity for this marker as follows: 45% for EPCs and 15% for BEPCs. Moreover, SSEA-4 expression was confirmed by ddPCR for EPCs (61%) and BEPCs (47%). Even though a partial expression (25%) was detected only in BEPCs by flow cytometry (Figure 5).

### 3.3. Neurogenic Differentiation of the Endothelial Progenitor Cells

**Figure 6:**
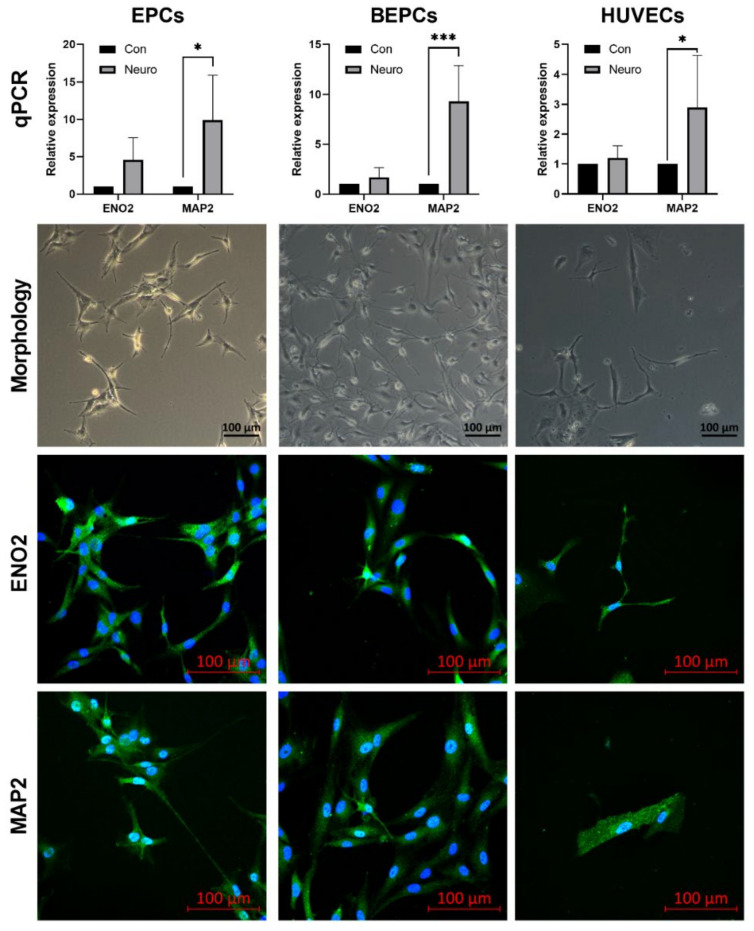
Neurogenic induction of rabbit and human endothelial progenitor cells. After induction, the neuron-like cells can be observed in the culture of all cell lines with the typical formation of axon- and dendrite-like cellular structures (Line 2; Zeiss Axio Observer.Z1/7 microscope; magnification at 100× for morphology and at 200× for confocal microscopy; scale bar = 100 μm). Moreover, all induced cell lines expressed the neuronal markers (ENO2 and MAP2) as confirmed by laser scanning confocal microscope Zeiss LSM 700 (Line 3 and 4; magnification at 200×; scale bar = 100 μm) or qPCR (Line 1). Blue—cell nuclei stained with DAPI, green—antibody staining; Con—control (uninduced) cells; Neuro—cells after neurogenic induction. The data are expressed as the mean ± SD; *—difference is statistically significant at *p* < 0.05; ***—difference is statistically significant at *p* < 0.001.

Morphological changes in both rabbit cell lines (EPCs and BEPCs) as well as in HUVECs were observed after 3 days of neurogenic induction (Figure 6). Neuron-like induced cells exhibited small nuclei bodies with clear halos and enhanced transparency. Moreover, cytoplasm was retracted towards the nucleus and simple or even branched axon-like processes were clearly visible in the cultures. The confocal fluorescent microscopy revealed an expression of neuronal markers ENO2 and MAP2 in all induced cell lines. In addition, qPCR analysis confirmed significantly increased relative expression of MAP2 in the induced BEPCs (*p* < 0.001) as well as in EPCs and HUVECs (*p* < 0.05) compared to uninduced cell cultures. Similarly, the relative expression of ENO2 in all induced cell lines increased when compared to uninduced cells, although not significantly (Figure 6).

### 3.4. Cryopreservation of the Rabbit EPCs and BEPCs

**Figure 7:**
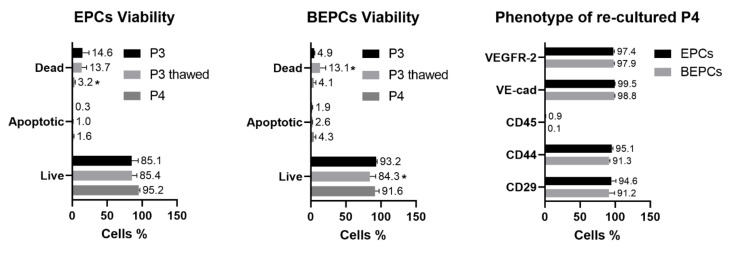
Viability and phenotype of the cryopreserved and re-cultured rabbit EPCs and BEPCs. P3—third passage of cells before freezing, P3 thawed—cryopreserved and thawed cells, P4—subsequent passage of the thawed and re-cultured cells. The data are expressed as the mean ± SD; *—difference is statistically significant at *p* < 0.05.

The viability of the thawed rabbit EPCs was not affected by the cryopreservation, since the proportion of live cells was similar as for the cells before freezing (P3) or after their subsequent culture (P4; Figure 7). Moreover, the proportion of apoptotic cells remained negligible (<2%) independent of the cryopreservation or subsequent culture. The proportion of dead cells even significantly (*p* < 0.05) decreased in the subsequent culture of the thawed EPCs (P4). In case of the rabbit BEPCs, cryopreservation significantly (*p* < 0.05) decreased the proportion of live cells and increased (*p* < 0.05) the number of dead cells in comparison to the cell viability before freezing (P3; Figure 7). However, the live cells of both rabbit cell lines still represented more than 84% of the thawed cells. In addition, their viability in the subsequent cultures (P4) reached the values of fresh cells before freezing (P3), thus confirming their good proliferation ability. Furthermore, those cells remained their endothelial phenotype in the subsequent passages of both, rabbit EPCs and BEPCs, which were highly (>90%) positive for CD29, CD44, VE-cadherin and VEGFR-2 and negative for CD45.

## 4. Discussion

The endothelial progenitor cells are a specific subpopulation of hematopoietic stem cells that are able to differentiate into the mature cells of endothelial lineage [5]. They originate in the bone marrow and are mobilized and circulated in the peripheral blood in order to repair the impaired endothelium and to generate the new blood vessels [53]. There has been enormous interest in the clinical or veterinary research of EPCs due to their outstanding regenerative potential. However, these cells might be also a valuable source of genetic information for the preservation of endangered animal species.

Here, we presented an optimized methodology for the isolation and adequate characterization of rabbit endothelial progenitor cells derived from peripheral blood (EPCs) and bone marrow (BEPCs) with respect to their typical morphology, phenotype, differentiation potential and cryopreservation. The morphology of rabbit EPCs and BEPCs cultured for less than 2–3 weeks were stable during the culture with spindle-shaped appearance (Figure 1) that agreed with the previous studies [41,42,44]. The same shape of the early EPCs morphology originated from peripheral blood and/or bone marrow was observed for human [54], mouse [30], rat [31,32,33,35], goat [38] or chicken [39]. However, it has been demonstrated that longer culture (more than 3 weeks) of rabbit EPCs caused a change of morphology to cobblestone-like cells, although not in BEPCs culture [42,43]. Similar morphological changes referred to the late EPCs were noticed in human, dog, sheep or goat EPCs from peripheral blood [22,36,37,38,54], but even in the bone marrow-derived EPCs of mouse [30] and rat [31,33]. By contrast, chicken EPCs from bone marrow displayed a cobblestone pattern only after the induction with vascular growth endothelial factor [39], whereas goat bone marrow-derived EPCs did not change the spindle-shaped morphology over the longer culture [38]. The cobblestone appearance similar to the typical morphology of HUVECs analyzed in our study or as reported elsewhere was also observed in human EPCs from umbilical cord [55]. Since, the rabbit EPCs and BEPCs in this study were cultured only until the third passage (plus one additional passage after the cell thawing), the morphological changes of the cultured rabbit cells cannot be confirmed by this study. However, according to the morphological shape, both rabbit cell lines may be considered as early EPCs. Moreover, from the aforementioned information, it can be assumed that morphological changes of the in vitro cultured EPCs may depend on the length of culture, tissue of origin or even on the species alone and a proper physiological induction factor.

When comparing to HUVECs, rabbit EPCs and BEPCs showed a similar and quite rapid proliferation rate about 20–30 h of population doubling time independently of the assessed passage (Figure 1). The same doubling time of the primary HUVECs can be found elsewhere. By contrast, human peripheral blood-derived early EPCs exhibited a lower proliferation rate than the late EPCs (about 34 h of doubling time) [54]. In addition, the doubling time of human umbilical cord-derived EPCs was calculated to be about 40 h [55]. The population doubling times of rat bone marrow-derived EPCs starting at 104 h (P0) decreased with the subsequent passages to 54 h (P4) and up to 40 h at passages 11 and 12 [35]. Thus, it seems that the proliferation rate of cultured EPCs may increase in the later passages. Although no differences in the proliferation of rabbit EPCs and BEPCs were observed here, the goat bone marrow-derived EPCs showed a remarkably longer doubling time (2.5 days) than the EPCs from peripheral blood (only 21 h for early and 31 h for late EPCs [38]. On the other hand, chicken bone marrow-derived EPCs reached a stable population doubling time ranged from 32 to 35 h (from passage 1 to 9) [39]. Similarly, doubling times of canine EPCs ranged between 23 and 29 h [36]. Hence, the proliferation rate of EPCs may depend on the biological source as well as on the species. Moreover, the rabbit cells cultured under the presented conditions maintained their viability over 80% during the whole culture with very low incidence of apoptosis (Table 3). However, an increased number of apoptotic cells was noticed in the rat bone marrow-derived late EPCs when incubated in the same medium as in our study [33]. However, this might be caused by the differentiated status of the cultured cells. Another study, which used the same rat cells, but different culture medium, did not observe a significant effect of the passage number on the cell viability [35].

In a previous study [42], a strong positivity for CD31 and vWF was observed for rabbit EPCs and HUVECs, however rabbit BEPCs did not express CD31. Moreover, cells showed low fluorescent signals for CD34 and CD45. After 4 weeks of culture, rabbit EPCs exhibited increased expression of VEGFR-2, while expression of α-SMA decreased in comparison to BEPCs. However, we found same strong and stable expression of VEGFR-2 and vWF in both rabbit and human cell lines (Table 4 and Table 5), and a significant decrease in CD31 expression was observed in both rabbit cell lines as analyzed by flow cytometry and qPCR (Table 4; Figure 2 and Figure 3) as well as confirmed by ddPCR in passage 3 (Figure 4 and Figure 5). On the other hand, HUVECs were highly positive for CD31 in our study. The contradictory results for the rabbit cells might be explained by the fact that latter passages (P5-P8; late EPCs) were analyzed in the aforementioned study, whereas cells at passage 3 were assessed here. Thus, the rabbit late EPCs may also co-expressed CD31, although the early one did not. Concerning the α-SMA, we noticed stable and high expression in both early rabbit cells, EPCs and BEPCs (Table 5).

The other studies on rabbit EPCs from peripheral blood demonstrated uptake of AcLDL and expression of VEGFR-2 after 7 days of culture [41,43], what agree with our results. However, one of those studies showed that rabbit cultured cells exhibited also the expression of CD34 marker when analyzed by immunofluorescence [41]. In addition, a different study also noticed CD34 expression (58%) as well as the expression of CD106 (64%) and CD146 (29%) after a week of culture. After two weeks, other markers were observed to be positive in rabbit cells, CD105 and VEGFR-2 [44]. By contrast, we did not detect any CD34 expression by flow cytometry in both rabbit cell lines (Table 4), though a different antibody was used as in the previously mentioned study. Unfortunately, at the present, there are not available anti-rabbit antibodies for the specific detection of rabbit CD34 as we have already discussed in our previous study [46]. Anyway, qPCR detected a decreasing tendency for CD34 expression with the following passages (P0–P3; Figure 2 and Figure 3) in both studied rabbit cell lines. Furthermore, ddPCR confirmed the negative expression of this marker for rabbit cells in P3 (Figure 4 and Figure 5). Similarly, HUVECs did not express CD34 as analyzed by flow cytometry. Moreover, it has been already published that the expression of CD34 disappeared in HUVECs cultured in vitro after several passages [56]. In comparison to the aforementioned rabbit studies, we similarly found the expression of CD105 in both rabbit cells as confirmed by flow cytometry and both PCR methods, although by two-fold lower than the expression in HUVECs. On the other hand, a weak and unstable expression of CD146 by rabbit EPCs and BEPCs was demonstrated by different methods, although HUVECs were strongly positive. This finding does not agree with the previous rabbit study [44], however a different antibody was used for the cell staining and no other method to confirm the marker expression was applied in that study.

Since a limited number of markers were analyzed in the rabbit studies, additional phenotype of rabbit EPCs and BEPCs have to be compared with works performed on different species. Murine EPCs demonstrated an AcLDL uptake after 7 days and expressed VEGFR-2, Sca-1 and CD133 after two weeks of culture [30]. Several studies assessed the phenotype of rat EPCs from bone marrow. The primary cells (P0) were positive (>30%) for CD29, CD45, CD90 and CD133, while expression of other markers (CD14, CD31, CD34, VE-cadherin and VEGFR-2) was low (<15%) [34]. Another study showed a high expression levels of CD31, CD34, CD133 and VEGFR-2 in rat EPCs cultured for 10 days, which obviously increased in comparison to the phenotype of freshly isolated bone marrow cells [32]. A stable expression of CD31, CD34 and VEGFR-2 was, thereafter, noticed in rat EPCs when passages 7 and 12 were compared, whereas a decrease in CD133 expression was observed in the latter mentioned passages [35]. AcLDL uptake was noticed in the late EPCs too, which were also positive for CD29, CD31, VE-cadherin, VEGFR-2, vWF and eNOS, while negative for CD45 [31,33]. In case of canine peripheral blood-derived EPCs, one study demonstrated the positive uptake of AcLDL, expression of VEGFR-2 and vWF, and the production of eNOS [36]. The other study comparing the phenotype of 1-week and 3-weeks old culture of EPCs, revealed a marked decrease in CD34 expression, but a notable increase in the expression of vWF and eNOS, while a weak or no expression of VEGFR-2 and CD146 was noticed [37]. The late EPCs derived from the peripheral blood of sheep also exhibited AcLDL uptake, expression of CD31, VEGFR-2 and vWF [22]. On the other hand, markable differences in the phenotype of goat EPCs were observed. Both EPCs lines, peripheral blood or bone marrow-derived, were able to incorporate AcLDL after one week of culture, however the latter mentioned were negative for CD31 and vWF. Whereas early as well as late EPCs from peripheral blood positively expressed the aforementioned markers [38]. The chicken bone marrow-derived EPCs exhibited a stable phenotype of CD34, CD133 and VEGFR-2 expressed markers in passages 1, 5 and 9. Similarly, in all passages an uptake of AcLDL was observed [39].

In our study, the expression of CD14 and CD45 significantly decreased with the following passages (Table 4) in both, rabbit EPCs and BEPCs. It has been reported that human early EPCs are positive for CD14 and CD45 markers, although their expression disappeared in late EPCs [3,25,54]. Similarly, HUVECs, a type of differentiated endothelial cells, did not express CD14. However, another reason of the reduced expression of CD14 and CD45 in rabbit cultures may be their purifying by the depletion of hematopoietic cells due to the medium changing and cell passaging. On the other hand, rabbit EPCs and BEPCs strongly expressed CD29 and CD44, that agree with HUVECs in case of CD29 (Table 4; Figure 4 and Figure 5). Moreover, expression of both markers, which belong to the family of cell adhesion molecules (CAMs), have been reported in several endothelial cell lines including HUVECs [57,58,59,60,61]. Additionally, rabbit EPCs and BEPCs as well as HUVECs strongly expressed also CD49f (Table 4). This marker was detected in various types of the human stem cells [62]. Furthermore, similar expression was also observed in other types of rabbit stem and progenitor cells analyzed in our laboratory (data not yet published). Hence, CD49f can be a valuable marker for EPCs phenotyping.

Other endothelial-associated markers, such as CD73, CD90 and CD105, have been expressed by rabbit EPCs and BEPCs in higher or lower levels as confirmed by flow cytometry and ddPCR (Table 4; Figure 4 and Figure 5). The analysis of these markers in rabbit cells by flow cytometry has been a big challenge due to the lack of information about the antibodies used in published papers [2]. HUVECs also exhibited a high expression of CD73 and CD105, though CD90 is expressed to a low degree. The expression of CD73 was reported previously in endothelial cells including HUVECs [63]. High expression of CD90 was detected in activated endothelial cells e.g., HUVECs, but not in non-activated cells [64]. However, rat EPCs expressed CD90 [34] similar as the rabbit cells in our study. We have already discussed the expression of CD105 above. By contrast, a very weak or hardly noticeable expression of CD133 was detected in both studied rabbit cell lines, whereas the aforementioned early EPCs from diffident animal species [34,39] or human [3] expressed this marker. VE-cadherin and VEGFR-2 are commonly reported markers for EPCs in above mentioned studies, thus their strong expression by rabbit endothelial cell lines was expected (Table 4). On the other hand, we did not notice the expression of CD166 by flow cytometry (Table 4), mainly due to the unspecificity of used antibody [2]. However, PCR techniques revealed expression of this marker, and much more than that, ddPCR allowed us to quantify this expression in rabbit EPCs as well as in BEPCs (Figure 4 and Figure 5). CD166 (ALCAM) belongs to CAMs family and was reported to be expressed by activated monocytes and endothelial cells [65]. Moreover, this marker is significantly associated with endothelial development [66]. Similarly, SSEA-4 was weakly detected in rabbit EPCs by flow cytometry (Table 4), although higher, but decreasing expression was noticed in rabbit BEPCs. On the other hand, qPCR observed stable expression of SSEA-4 synthase (ST3GAL2), an indicator of SSEA-4 synthesis [2], and ddPCR successfully quantified its expression in both rabbit cell lines (about 50%; Figure 4 and Figure 5). SSEA-4 has been also detected in the human EPCs from different sources [67]. MSCA-1, referred to a non-specific alkaline phosphatase (ALPL), was reported in human [68] and rabbit [2] mesenchymal stem cells. However, as for CD133, a weak or hardly noticeable ALPL expression was noticed in both rabbit cell lines as confirmed by flow cytometry as well as by PCR methods. Anyway, mouse EPCs were also negative for alkaline phosphatase [69]. In addition, higher levels of ALPL were observed only in EPCs, which were simultaneously cultured with mesenchymal stem cells in rabbit [42] or goat [38].

Uptake of AcLDL is one of the most common reported endothelial features in the majority of published works or here mentioned studies focused on EPCs. Beside AcLDL endocytosis and stable expression of endothelial-associated intracellular markers (vWF and eNOS) by rabbit cells and HUVECs, also expression of common cytoskeletal markers (vimentin, desmin and α-SMA) was observed in both, rabbit EPCs and BEPCs (Table 5). Vimentin was reported to be found in the mesenchymal as well as endothelial cells [70]. Desmin occurs mainly in muscle and endothelial cells [71]. However, we found this protein also in rabbit mesenchymal stem cells [2,47]. Moreover, it was reported that endothelial cells derived from CD34^+^ human cord blood and HUVECs expressed beside the endothelial-associated markers also α-SMA [72]. Recently, aldehyde dehydrogenase (ALDH) has been reported as a reliable stem cell marker already detected in over 80 different tissues or cell types using an aldefluor assay by flow cytometry. We have already proved the expression of ALDH by chicken primordial germ cells [48] or by rabbit mesenchymal stem cells of different biological origin (data not yet published). Similarly, here we found a stable and relatively high ALDH expression in rabbit EPCs, while lower levels of ALDH was observe in rabbit BEPCs (Table 5; Figure 4 and Figure 5). These findings are in agreement with another study, where an ALDH activity was confirmed in human EPCs [73]. Although, a strong ALDH activity was also monitored in HUVECs here, as far we know, no other published work studied the expression of ALDH in HUVECs. However, one study reported a low ALDH activity in normal endothelial cells isolated from the mouse dermis, while a slight increased expression was observed in tumor endothelial cells [74].

In summary, both rabbit endothelial progenitor cells (EPCs and BEPCs at P3) possess very similar and stable phenotype: CD14^−^CD29^+^CD31^−^CD34^−^CD44^+^CD45^−^CD49f^+^CD73^+^CD90^+^CD105^+^CD133^−^CD146^−^CD166^+^VE-cadherin^+^VEGFR-2^+^SSEA-4^+^MSCA-1^−^vWF^+^eNOS^+^AcLDL^+^ALDH^+^vimentin^+^desmin^+^α-SMA^+^. According to the above-mentioned published studies, we can conclude, that these rabbit cells express markers typical for early EPCs, although some differences can be noticed among rabbit studies as well as in comparison to other animal studies. However, a variability in the phenotype of human early EPCs was also reported, since according to different studies they were CD14^+/-^CD31^+/-^CD34^+^CD45^+^CD133^+/-^VE-cadherin^+/-^VEGFR-2^+/-^vWF^+^eNOS^+^AcLDL^+^ [3]. The hypothesis, that early rabbit EPCs were analyzed here, can be supported by the expression of slightly different phenotype observed in HUVECs (CD14^−^CD29^+^CD31^+^CD34^−^CD49f^+^CD73^+^CD90^+/low^CD105^+^CD133^−^CD146^+^VE-cadherin^+^VEGFR-2^+^SSEA-4^−^vWF^+^eNOS^+^AcLDL^+^ALDH^+^), where markers referred to late EPCs (CD31 and CD146) were detected. Recently was demonstrated that human late EPCs possess similar phenotype (CD31^+^CD34^−^CD45^−^CD90^−^CD133^−^CD146^+^VEGFR-2^+^vWF^+^) as HUVECs [26]. Hence, although the flow cytometry or immunofluorescence alone is a standard method for the cell phenotyping, inclusion of other methods such as ddPCR is crucial for discovering the true cell phenotype. However, as those methods are based on distinct molecular principles, they cannot be comparable, and should be used in a combination with each other.

Since neuron-like transdifferentiation ability was observed in HUVECs [27,28,29], we performed a neurogenic induction in both rabbit cell lines as well as in HUVECs using a commercial kit. Typical morphological changes reported for the induced neuron-like cells [52,75,76] were monitored already after 3 days of induction in rabbit and human endothelial cultures. Moreover, expression of neuronal marker (ENO2 and MAP2) by the induced cells was confirmed using confocal microscopy and qPCR (Figure 6). As far we know, this is the first study reported a neuron-like differentiation potential of rabbit EPCs. On the other hand, transdifferentiation of neuronal stem cells to endothelial cells has been also reported, thus suggesting the existence of common progenitor shared by both cell types [3].

In order to maintain the highest quality of the cryopreserved cells, an optimal freezing protocol has to be used. Here, a standard methodology for the cryopreservation of rabbit mesenchymal stem cells [2] was applied also for rabbit endothelial cells with a slight modification as described in the Methods. The thawed rabbit cells from both biological sources maintained high viability and low apoptosis rate (Figure 7) even after 3 months of storage in liquid nitrogen. Moreover, after a few days in culture (P4), the confluent cells still expressed endothelial-associated markers CD29, CD44, VE-cadherin and VEGFR-2 significantly. Similarly, the cryopreservation had no effect on the viability and phenotype of rat or chicken EPCs [35,39].

## 5. Conclusions

Here, we proposed a methodology for the isolation and expansion of early EPCs under the optimized culture conditions that maintain the viability and proliferation rate of rabbit endothelial progenitor cells derived from peripheral blood and bone marrow. The true phenotype of rabbit early EPCs was determined by the combination of standard laboratory methods (flow cytometry and qPCR). Moreover, a novel ddPCR method allowed us to quantify the expression of those markers that cannot be properly analyzed using flow cytometry due to the lack of specific antibodies. In addition, both rabbit cell lines exhibited a neuron-like differentiation potential that can be further studied. The viability and phenotype of rabbit EPCs were not affected by the cryopreservation, thus making them a valuable source of primitive cells for animal gene banks. In conclusion, our findings suggest that cells expanded from the rabbit peripheral blood and bone marrow are of the endothelial origin with a stable marker expression and interesting proliferation and differentiation capacity. However, a further study is required in order to assess the morphological and phenotypical changes in the rabbit culture of late EPCs.

## Figures and Tables

**Table 1:** List of primary antibodies used for flow cytometry.

Marker	Host/Isotype	Clone	Conjugate	Company
CD14	mouse IgG2a	TÜK4	PE	Dako Cytomation
CD29	mouse IgG1	P4G11	FITC	Merck
CD31	mouse IgG1	C31.7	PE	Novus Biologicals
CD34	mouse IgG1	QBEnd-10	FITC	Thermo Fisher Scientific
CD44	mouse IgG1	W4/86	purified ^1^	Bio-Rad
CD45	mouse IgG1	L12/201	purified ^1^	Bio-Rad
CD49f	rat IgG2a	GoH3	AF647	BioLegend
CD73	mouse IgG1	AD2	FITC	BD Biosciences
CD90	mouse IgG1	5E10	FITC	BD Biosciences
CD105	mouse IgG1	266	FITC	BD Biosciences
CD133	mouse IgG1	AC133	PE	Miltenyi Biotec
CD146	mouse IgG1	P1H12	PE	eBioscience
CD166	rabbit IgG	polyclonal	purified ^1^	Bioss
VE-cadherin	mouse IgG1	F-8	AF647	Santa Cruz Biotechnology
Flk-1 (VEGFR-2)	mouse IgG1	D-8	AF647	Santa Cruz Biotechnology
SSEA-4	mouse IgG3	MC-813-70	PE	eBioscience
MSCA-1	mouse IgG1	W8B2	PE	Miltenyi Biotec
Vimentin	mouse IgG2a	Vim 3B4	purified ^1^	Dako Cytomation
Desmin	mouse IgG1	D33	purified ^1^	Dako Cytomation
α-SMA	mouse IgG2a	1A4	purified ^1^	Dako Cytomation
vWF	mouse IgG1	C-12	FITC	Santa Cruz Biotechnology
NOS3 (eNOS)	mouse IgG2a	A-9	FITC	Santa Cruz Biotechnology

^1^ Cells stained with the purified antibodies were subsequently incubated with proper secondary antibodies.

**Table 2:** Gene-specific primers and size of polymerase chain reaction (PCR) products.

Gene	Product Size (bp)	Forward Primer	Reverse Primer	Reference
CD29	287	5´-AGAATGTCACCAACCGTAGCA-3´	5´-CACAAAGGAGCCAAACCCA-3´	[2]
CD31	138	5´-GTGATAATTGCCGCCTTGAT-3´	5´-GTTGGGATCTGACACGGTCT-3´	XM_008271715.2 ^1^
CD34	155	5´-CTGAGGTTAGGGCTCAGTGC-3´	5´-GGAGTAGCTCTGGTGGCTTG-3´	[50]
CD44	112	5´-TCATCCTGGCATCCCTCTTG-3´	5´-CCGTTGCCATTGTTGATCAC-3´	[2]
CD45	262	5´-TACTCTGCCTCCCGTTG-3´	5´-GCTGAGTGTCTGCGTGTC-3´	[2]
CD73	170	5´-CTCCTTTCCTCTCAAATCCAG-3´	5´-GTCCACGCCCTTCACTTTC-3´	[2]
CD90	293	5´-CTGCTGCTGCTCTCACTGTC-3´	5´-ACAGAAGCAGCTTTGGGAAA-3´	[2]
CD105	109	5´-TGACATACAGCACCAGCCAG-3´	5´-AGCTCTGACACCTCGTTTGG-3´	[2]
CD133	124	5´-TCATTCCGATGGAACAGTCA-3´	5´-ATGAAGTTCTGGGCGTCATC-3´	XM_017350443.1 ^1^
CD146	116	5´-GACAATGGCATCCTGGTCTT-3´	5´-AGTAGCTCGTGGCGTTCACT-3´	[2]
CD166	345	5´-GCTCCCCAGTATTTATTGCCTTC-3´	5´-GTAGCACCTTTCCATTCCTGTA-3´	[2]
ST3GAL2 (SSEA-4)	126	5´-CTGGGAGAATAACCGGTACG-3´	5´-GCTCAGTTGCCTCGGTAGAC-3´	[2]
ALPL (MSCA-1)	137	5´-CCCTCATGTGATGGCTTACG-3´	5´-CTCAGAACAGGACGCTCAGG-3´	[2]
ALDH	135	5´-CTGGGAAAAGCAACCTGAAG-3´	5´-AACACTGGCCCTGATGGTAG-3´	AB176450.1 ^1^
B2M	118	5´-ATTCACGCCCAATGATAAGG-3´	5´-ATCCTCAGACCTCCATGCTG-3´	[2]

^1^ NCBI Reference Sequence.

**Table 3:** Viability analysis of different passages of rabbit endothelial progenitor cells derived from two biological sources.

Cell Status /Passage	Cell Type	P0	P1	P2	P3
Live	EPCs	83.8 ± 4.8	84.7 ± 1.3	91.5 ± 2.9	85.1 ± 9.3
	BEPCs	93.0 ± 3.3	89.2 ± 6.7	92.5 ± 2.8	93.2 ± 1.3
Apoptotic	EPCs	0.9 ± 0.6	1.0 ± 0.8	0.6 ± 0.1	0.3 ± 0.1
	BEPCs	1.0 ± 0.4	1.3 ± 0.9	2.1 ± 0.9	1.9 ± 0.8
Dead	EPCs	15.4 ± 4.6	14.2 ± 1.9	7.9 ± 3.0	14.6 ± 9.4
	BEPCs	6.0 ± 3.1	9.5 ± 5.9	5.5 ± 2.1	4.9 ± 0.9

The data are expressed as the mean ± SD; P0—initial culture, P1—first passage, P2—second passage, P3—third passage; Live—unstained cells; Apoptotic—cells positive for Annexin V; Dead—cells positive for propidium iodide.

**Table 4:** Surface marker expression of rabbit and human endothelial cells analyzed by flow cytometry.

Marker/Passage	Cell Type	P0	P1	P2	P3	HUVECs (P3)
CD14	EPCs	8.0 ± 7.9	2.0 ± 2.3 *	0.9 ± 0.7 **	1.2 ± 0.9 **	0.1 ± 0.0
	BEPCs	7.5 ± 6.4	1.1 ± 1.0 *	0.1 ± 0.1 **	0.1 ± 0. 0 **	
CD29	EPCs	84.3 ± 9.1	90.7 ± 6.4	94.3 ± 6.3 *	95.2 ± 4.3 **	91.2 ± 0.3
	BEPCs	81.1 ± 4.0	93.5 ± 7.6	89.4 ± 11.4	91.8 ± 4.3	
CD31	EPCs	11.6 ± 9.0	4.3 ± 3.9 *	2.0 ± 1.2 **	1.1 ± 0.4 **	81.5 ± 1.2
	BEPCs	19.8 ± 8.9	7.7 ± 1.2 **	4.7 ± 2.5 ***	1.8 ± 1.0 ***	
CD34	EPCs	0.8 ± 0.3	0.6 ± 0.3	0.4 ± 0.3	0.5 ± 0.2	0.6 ± 0.6
	BEPCs	0.7 ± 0.5	0.4 ±0.2	0.1 ± 0.1	0.1 ± 0.1	
CD44	EPCs	83.6 ± 12.2	85.9 ± 13.5	96.5 ± 2.2	94.6 ± 4.7	n/a
	BEPCs	96.7 ± 1.9	96.6 ± 4.3	96.9 ± 1.0	94.2 ± 3.9	
CD45	EPCs	10.1 ± 9.1	3.0 ± 3.4 **	1.8 ± 2.4 **	1.3 ± 1.1 ***	n/a
	BEPCs	15.7 ± 10.3	1.8 ± 1.1 **	0.3 ± 0.1 ***	0.2 ± 0.1 ***	
CD49f	EPCs	n/a	n/a	n/a	85.5 ± 6.8	94.1 ± 8.4
	BEPCs	n/a	n/a	n/a	85.8 ± 11.0	
CD73	EPCs	n/a	n/a	n/a	68.4 ± 11.5	91.0 ± 1.4
	BEPCs	n/a	n/a	n/a	71.3 ± 6.9	
CD90	EPCs	n/a	n/a	n/a	90.4 ± 9.82	12.6 ± 0.5
	BEPCs	n/a	n/a	n/a	88.1 ± 11.9	
CD105	EPCs	n/a	n/a	n/a	41.4 ± 6.0	83.7 ± 1.2
	BEPCs	n/a	n/a	n/a	33.0 ± 21.2	
CD133	EPCs	0.7 ± 0.3	0.4 ± 0.3	0.3 ± 0.2	0.5 ± 0.5	0.5 ± 0.2
	BEPCs	1.2 ± 1.2	0.4 ± 0.2	0.2 ± 0.1	0.3 ± 0.4	
CD146	EPCs	2.3 ± 1.8	2.0 ± 2.3	2.6 ± 3.8	6.3 ± 7.1	83.7 ± 18.5
	BEPCs	1.5 ± 1.1	3.9 ± 1.9 *	0.9 ± 0.8	0.3 ± 0.4	
CD166	EPCs	0.2 ± 0.2	0.1 ± 0.0	0.1 ± 0.0	0.1 ± 0.0	n/a
	BEPCs	n/a	n/a	n/a	n/a	
VE-cadherin	EPCs	94.8 ± 4.3	98.1 ± 1.2 *	98.8 ± 1.0 **	98.4 ± 1.7 *	84.3 ± 12.7
	BEPCs	99.1 ± 0.4	99.8 ± 0.1	99.8 ± 0.2	96.0 ± 6.7	
VEGFR-2	EPCs	90.2 ± 8.2	94.5 ± 8.6	97.1 ± 1.8	97.4 ± 2.3	94.8 ± 5.3
	BEPCs	97.8 ± 2.0	99.4 ± 0.3	99.2 ± 0.5	94.1 ± 9.0	
SSEA-4	EPCs	6.2 ± 5.2	5.7 ± 10.1	1.4 ± 1.1	1.0 ± 0.7	1.0 ± 0.1
	BEPCs	59.6 ± 29.7	19.5 ± 11.8 *	14.3 ± 9.7 **	25.4 ± 18.0 *	
MSCA-1	EPCs	0.5 ± 0.4	0.4 ± 0.3	0.1 ± 0.2	0.2 ± 0.1	n/a
	BEPCs	1.0 ± 0.5	0.3 ± 0.2	0.0 ± 0.0	0.1 ± 0.0	

The data are expressed as the mean ± SD; P0—initial culture, P1—first passage, P2—second passage, P3—third passage; *—difference is statistically significant at *p* < 0.05; **—difference is statistically significant at *p* < 0.01.; ***—difference is statistically significant at *p* < 0.001 in comparison to P0; n/a—not analyzed.

**Table 5:** Intracellular marker expression of rabbit and human endothelial cells analyzed by flow cytometry.

Marker/Passage	Cell Type	P0	P1	P2	P3	HUVECs (P3)
AcLDL	EPCs	80.8 ± 10.8	92.8 ± 7.8	88.7 ± 13.9	92.2 ± 5.4	86.2 ± 1.0
	BEPCs	99.3 ± 0.5	99.9 ± 0.2	98.8 ± 1.0	97.6 ± 0.9 **	
ALDH	EPCs	84.9 ± 5.6	78.9 ± 10.3	80.4 ± 15.4	94.5 ± 5.0	80.0 ± 1.8
	BEPCs	24.7 ± 4.9	46.1 ± 25.2	48.6 ± 40.4	25.3 ± 10.5	
Vimentin	EPCs	81.3 ± 5.9	87.3 ± 7.7	89.6 ± 7.3	86.3 ± 9.0	n/a
	BEPCs	99.3 ± 0.3	98.0 ± 1.5	99.4 ± 0.5	99.0 ± 0.7	
Desmin	EPCs	55.1 ± 18.2	59.0 ± 17.9	68.3 ± 20.2	64.2 ± 14.0	n/a
	BEPCs	89.5 ± 5.8	92.3 ± 6.0	94.8 ± 3.4	92.4 ± 5.4	
α-SMA	EPCs	74.0 ± 5.9	85.6 ± 11.1	88.5 ± 4.9	82.5 ± 9.9	n/a
	BEPCs	99.0 ± 0.3	96.9 ± 2.0	98.1 ± 1.1	92.7 ± 10.4	
vWF	EPCs	98.0 ± 0.1	98.3 ± 0.8	99.4 ± 0.3 *	99.8 ± 0.1 **	98.8 ± 1.5
	BEPCs	97.4 ± 1.4	99.5 ± 0.3 **	99.7 ± 0.2 ***	99.8 ± 0.1 ***	
eNOS	EPCs	94.8 ± 6.0	99.0 ± 0.3	99.4 ± 0.2	99.7 ± 0.1	98.9 ± 1.0
	BEPCs	99.8 ± 0.1	99.9 ± 0.1	99.9 ± 0.1	99.9 ± 0.1	

The data are expressed as the mean ± SD; P0—initial culture, P1—first passage, P2—second passage, P3—third passage; AcLDL—acetylated low-density lipoprotein; ALDH—aldehyde dehydrogenase; α-SMA—α-smooth muscle actin; vWF—von Willebrand factor; eNOS—endothelial nitric oxide synthase; *—difference is statistically significant at *p* < 0.05; **—difference is statistically significant at *p* < 0.01.; ***—difference is statistically significant at *p* < 0.001 in comparison to P0; n/a—not analyzed.

## Data Availability

The data presented in this study are available in article.

## Appendix B

**Table A1:** Comparative homology of rabbit and human markers analyzed by flow cytometry using antibodies cross-reactive to different species.

Marker	Species	Homology (%)	NCBI Reference
CD14	Rabbit	73.07	NP_001075664.1
	Human	100	NP_000582.1
CD29 (ITB1)	Rabbit	100	AAB34684.1
	Human	100	AAA79835.1
CD31 (PECAM1)	Rabbit	73.99	XP_008269937.1
	Human	100	NP_000433.4
CD34	Rabbit	71.76	XP_008266693.1
	Human	100	NP_001020280.1
CD49f (ITGA6)	Rabbit	92.74	XP_017198420.1
	Human	100	NP_001073286.1
CD73 (NT5E)	Rabbit	89.39	XP_002714603.1
	Human	100	NP_002517.1
CD90 (THY1)	Rabbit	82.23	XP_002722764.1
	Human	100	NP_006279.2
CD105 (ENG)	Rabbit	72.84	XP_008249251.1
	Human	100	NP_001108225.1
CD133 (PROM1)	Rabbit	68.31	XP_017205932.1
	Human	100	NP_006008.1
CD146 (MCAM)	Rabbit	82.44	XP_008248732.2
	Human	100	NP_006491.2
CD166 (ALCAM)	Rabbit	95.20	XP_002716771.1
	Human	100	NP_001618.2
VE-cadherin (CDH5)	Rabbit	83.72	XP_008255604.2
	Human	100	NP_001786.2
VEGFR-2 (KDR)	Rabbit	87.54	NP_001182599.1
	Human	100	NP_002244.1
SSEA-4 (ST3GAL2)	Rabbit	98.29	XP_002711761.1
	Human	100	NP_008858.1
MSCA-1 (ALPL)	Rabbit	91.93	XP_017201978.1
	Human	100	NP_000469.3
Vimentin	Rabbit	96.78	XP_002717466.1
	Human	100	NP_003371.2
Desmin	Rabbit	98.51	NP_001164952.1
	Human	100	NP_001918.3
α-SMA (ACTA2)	Rabbit	100	NP_001095152.1
	Human	100	NP_001604.1
vWF	Rabbit	83.02	NP_001316017.1
	Human	100	NP_000543.3
eNOS (NOS3)	Rabbit	99.15	AAG24287.1
	Human	100	NP_000594.2
